# BTN3A2 Expression Is Connected With Favorable Prognosis and High Infiltrating Immune in Lung Adenocarcinoma

**DOI:** 10.3389/fgene.2022.848476

**Published:** 2022-07-06

**Authors:** Yuansheng Lin, Hao Zhou, Shengjun Li

**Affiliations:** Suzhou Science and Technology Town Hospital, Gusu School, Nanjing Medical University, Suzhou, China

**Keywords:** BTN3A2, prognosis, immune infiltration, LUAD, TCGA

## Abstract

**Background:** Butyrophilin subfamily 3 member A2 (BTN3A2) is an important mediator in immune activation, and it is reported to be linked to many cancer progresses. However, the relation with infiltrating immune and prognosis of BTN3A2 in lung adenocarcinoma are not clear.

**Methods:** In our study, we checked the mRNA expression and protein expression profile of BTN3A2 in lung adenocarcinoma (LUAD) and its relation to clinical outcomes using TIMER and UALCAN databases. In addition, we analyzed the survival of BTN3A2 in LUAD using the Kaplan–Meier Plotter database and PrognoScan database. Moreover, we analyzed gene set enrichment analysis (GSEA) of the BTN3A2. Next, we explored the relation of BTN3A2 expression with the immune infiltration by TIMER. At last, in order to enrich the regulatory mechanism of BTN3A2, we used miRarbase, starbase, and miRDB databases to look for miRNA targets of BTN3A2.

**Results:** The mRNA along with the protein expression of BTN3A2 in the LUAD group was lower than that in the normal group. In addition, high BTN3A2 expression was connected with good first progression (FP) and overall survival (OS) in LUAD. Then, the GSEA analysis demonstrated that T-cell receptor signaling cascade, B-cell receptor signaling cascade, natural killer cell–mediated cytotoxicity, immune receptor activity, immunological synapse, and T-cell activation were enriched differentially in the BTN3A2 high expression phenotype of LUAD. Moreover, BTN3A2 expression is a remarkable positive correlation with invading levels of tumor purity, B cells, neutrophils, CD4+ T cells, dendritic cells, macrophages, and CD8+ T cells in LUAD, and B cells and dendritic cells were linked with a good prognosis of LUAD. To further enrich the possible regulatory mechanisms of BTN3A2, we analyzed the miRNA targets. The results showed that hsa-miR-17-5p may be miRNA targets of BTN3A2.

**Conclusion:** Taking together, we provide evidence of BTN3A2 as possible prognosis biomarkers of LUAD. In addition, high BTN3A2 expression in LUAD may influence the prognosis because of immune invasion. Moreover, our findings provide a potential mechanism that hsa-miR-17-5p may be miRNA targets of BTN3A2.

## Background

Lung cancer is the most frequent cause of cancer-linked deaths universally. A total of 1.8 million people are diagnosed with lung cancer and 1.6 million people die of the lung cancer each year (Hirsch et al., 2017). In spite of advances in therapy choices, such as irradiation, iatrochemistry, surgery, and targeted treatment, prognosis remains poor due to the presence of metastatic cancers in most patients at the moment of diagnosis (Chen et al., 2014; Altorki et al., 2019). Thus, finding appropriate prognostic biomarker is crucial for LUAD patients.

Butyrophilin (BTN) family members are immunoglobulin-like molecules, which act as immune checkpoint modulators and play a role in self tolerance. BTN3 family members include BTN3A1, BTN3A2, and BTN3A3. The expression of BTN3A2 in neonatal autoimmune and allergic diseases has a strong causal relationship (Huang et al., 2020). BTN3A2 expression is regulated by stress, which is linked to the prognosis of pancreatic cancer, and may constitute an immune escape mechanism to prevent Vγ 9Vδ2 T-cell recognition (Vantourout et al., 2018). Studies suggest that BTN3A2 is differentially expressed in a variety of cancers. BTN3A2 is expressed in triple-negative breast cancer, pancreatic ductal carcinoma, and epithelial ovarian cancer, and is associated with a good prognosis (Le Page et al., 2012; Cai et al., 2020). BTN3A2 is highly expressed and repressed the growth, migration, and infiltration of gastric cancer cells (Han et al., 2017; Zhu et al., 2017). Nevertheless, the role of BTN3A2 in LUAD is unclear.

Herein, we first explored the mRNA along with the protein expression of BTN3A2 in the LUAD, TIMER, and UALCAN databases. Then, we used the Kaplan–Meier Plotter database to analyze the survival of BTN3A2 in LUAD. Next, we utilized the UALCAN database and GSEA software to analyze the BTN3A2 promoter methylation level. Moreover, we used the TIMER database to explore the relationship of BTN3A2 expression with the immune infiltration. At last, we analyzed miRNA targets to enrich the possible regulatory mechanism of BTN3A2. The results showed that high BTN3A2 expression in LUAD may influence the prognosis because of immune invasion. Moreover, our findings provide a potential mechanism that hsa-miR-17-5p may be miRNA targets of BTN3A2.

## Results

### Butyrophilin Subfamily 3 Member A2 Expression Levels in Different Human Cancers and Lung Adenocarcinoma

**FIGURE 1:**
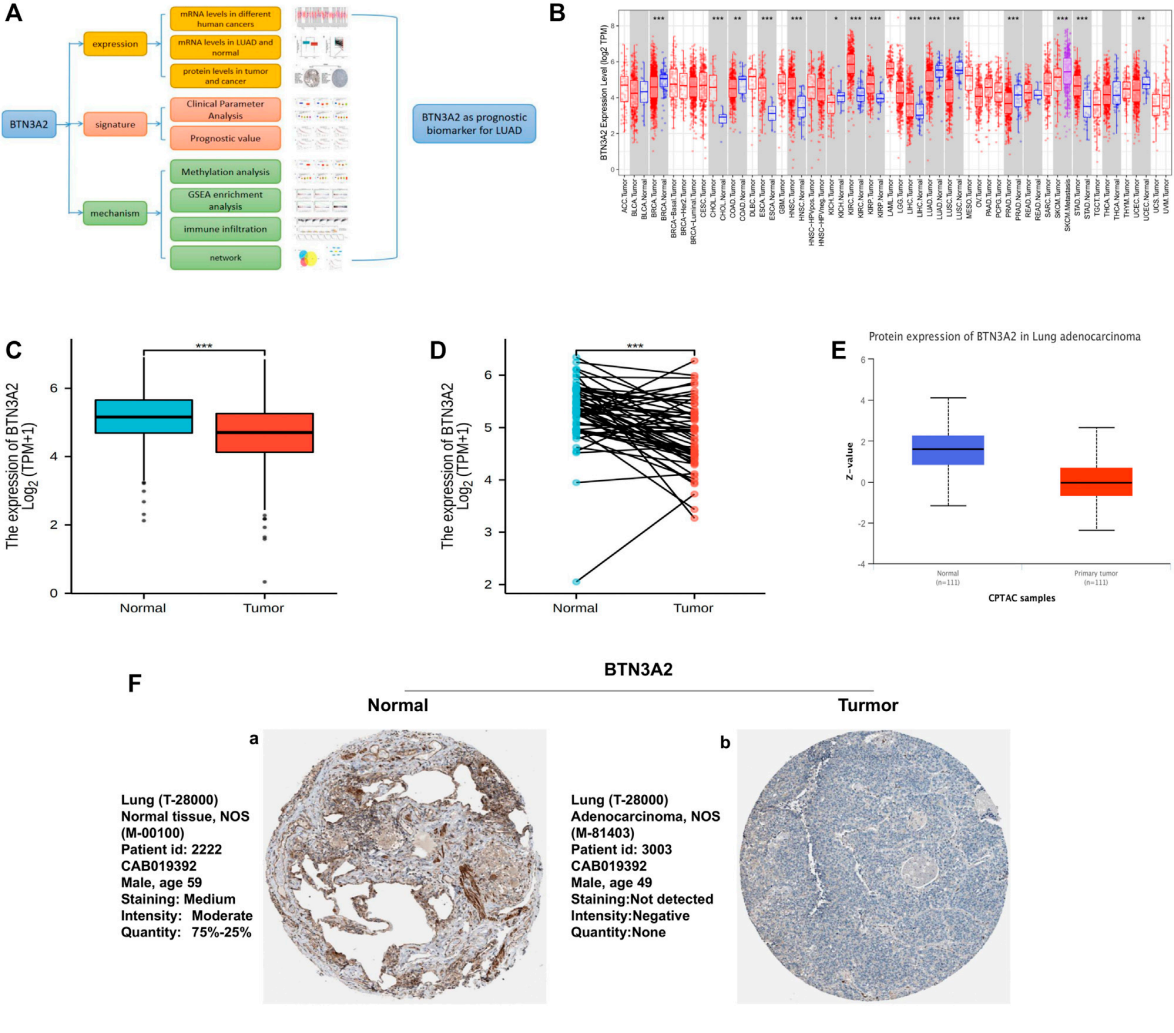
BTN3A2 contents in various human cancers and LUAD. **(A)** Workflow of this study. **(B)** BTN3A2 contents in different cancers from TCGA data resource were analyzed by TIMER. **(C)** BTN3A2 contents in non-malignant and tumor tissues of LUAD patients in the TCGA database. **(D)** BTN3A2 contents in pre-disease and post-disease form the same sample. **(E)** UALCAN database showed the protein expression of BTN3A2 in non-malignant and LUAD groups from CPTAC samples. **(F)** Expression of BTN3A2 in LUAD samples and normal tissues in the Human Protein Atlas.

The study design is shown as a flowchart for the analysis of the BTN3A2 gene (Figure 1A). To detect BTN3A2 expression in cancer and non-malignant tissues, the BTN3A2 mRNA contents in different cancer type tissues were analyzed using the Sangerbox and TIMER data resources. The results illustrated that the BTN3A2 expression was lower in LUAD, lung squamous cell carcinoma, as well as kidney renal clear cell carcinoma than that in the non-malignant tissues (Figure 1B). Moreover, data from TCGA demonstrated that the BTN3A2 content was lower in the LUAD group than that in the healthy group (Figures 1C,D). Interestingly, the protein expression of BTN3A2 in LUAD was still much lower than in that in non-malignant tissues from CPTAC samples (UALCAN) (Figure 1E). To verify the histological level of BTN3A2, we used the Human Protein Atlas database. The results showed that BTN3A2 is upregulated in normal tissue and downregulated in LUAD tissues (Figure 7F). The result was found in immunofluorescence staining (Supplementary Figure S1). Otherwise, BTN3A2 is also highly expressed in cells with high CD3 expression.

### Butyrophilin Subfamily 3 Member A2 Transcription in Subgroups of Patients With Lung Adenocarcinoma, Stratified Based on Gender, Race, Nodal Status Metastasis, and Other Criteria (UALCAN)

**FIGURE 2:**
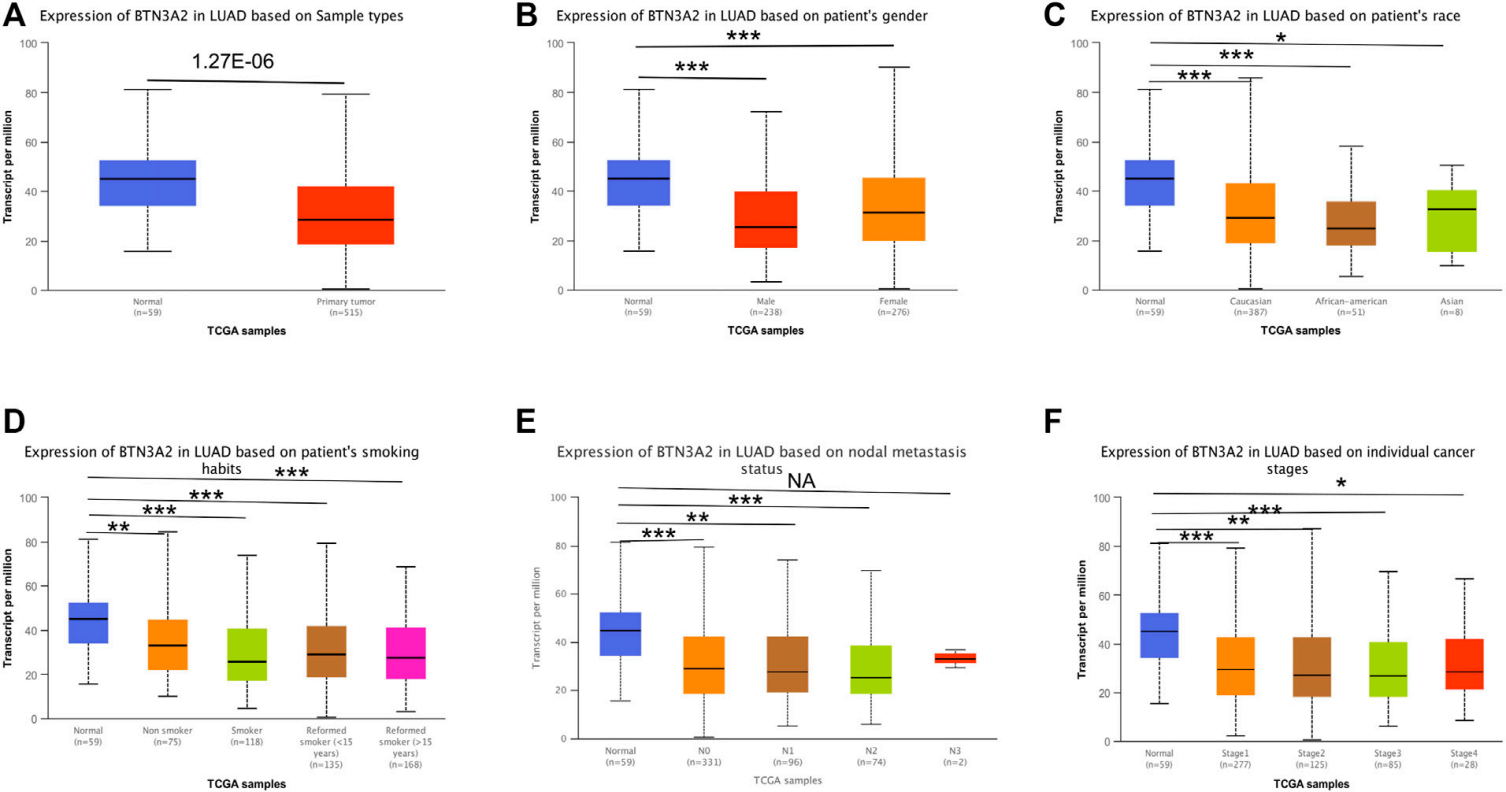
BTN3A2 contents in LUAD (UALCAN). **(A–F)** BTN3A2 contents were low in LUAD. (NA, *p* > 0.05, ∗, *p* < 0.05, ∗∗, *p* < 0.01, ∗∗∗, *p* < 0.001).

Further analysis of multiple clinic features of 515 LUAD samples in TCGA exhibited the low BTN3A2 mRNA content. The BTN3A2 mRNA content was lower in LUAD patients than that in healthy individuals in subgroup analysis on the basis of gender, race, nodal metastasis, smoking, stages, as well as tumor grade (Figure 2 and Table 1). Therefore, BTN3A2 levels may have a promising LUAD diagnostic value.

**TABLE 1:** Baseline characteristics of patients with LUAD.

Characteristic	Low expression of BTN3A2	High expression of BTN3A2	*p*
N	267	268	
T stage, n (%)			0.069
T1	76 (14.3%)	99 (18.6%)	
T2	151 (28.4%)	138 (25.9%)	
T3	31 (5.8%)	18 (3.4%)	
T4	9 (1.7%)	10 (1.9%)	
N stage, n (%)			0.564
N0	171 (32.9%)	177 (34.1%)	
N1	48 (9.2%)	47 (9.1%)	
N2	43 (8.3%)	31 (6%)	
N3	1 (0.2%)	1 (0.2%)	
M stage, n (%)			0.488
M0	183 (47.4%)	178 (46.1%)	
M1	15 (3.9%)	10 (2.6%)	
Age, median(IQR)	65 (58, 72)	67 (59, 73)	0.104

### Prognostic Potential of Butyrophilin Subfamily 3 Member A2 in Lung Adenocarcinoma

**FIGURE 3:**
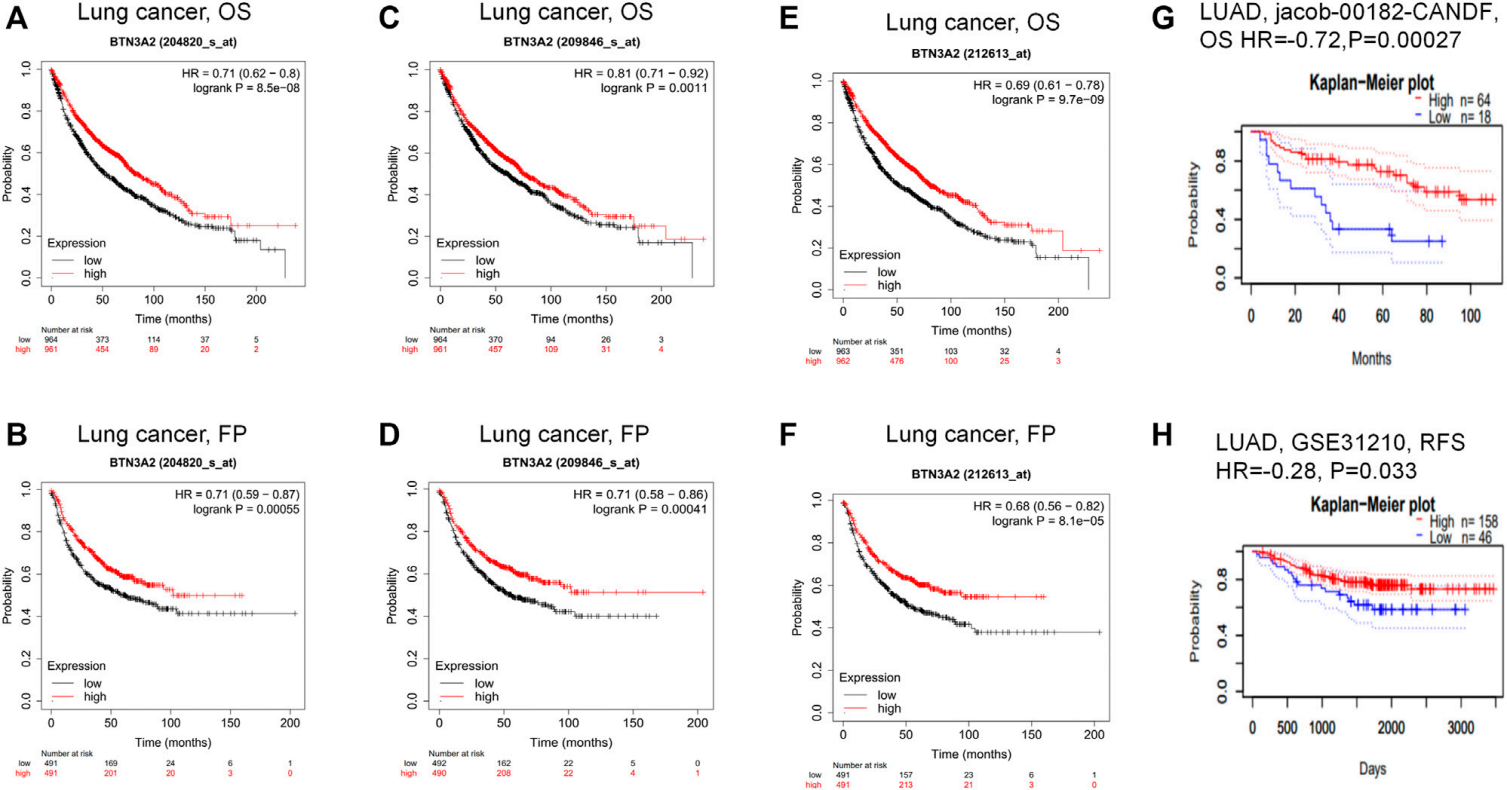
Comparing the high and low expression of BTN3A2 in LUAD by Kaplan–Meier survival curves. **(A–B)** OS and FP survival curves of lung cancer in 204820_s_at probe (*n* = 1295, *n* = 982). **(C–D)** OS and FP survival curves of lung cancer in 209846_s_at probe. (*n* = 1295, *n* = 982). **(E–F)** FP and OS survival curves of lung cancer in 212613_s_at probe (*n* = 1295, *n* = 982). **(G–H)** OS and RFS survival curves of LUAD in jacob-00182-CANDF and GSE31210, respectively. OS, overall survival; FP, first progression; RFS, relapse free survival.

To explore whether BTN3A2 expression was related to the prognosis of LUAD patients, we evaluated the influence of BTN3A2 contents on OS and FP through the Kaplan–Meier plotter data resource. The data demonstrated that the high BTN3A2 contents were linked to the good prognosis in LUAD from 204820_s_at probe (OS HR = 0.71, *p* = 8.5e-08; FP HR = 0.71, *p* = 5.5e-04) (Figures 3A,B). The results were consistent with those in 209846_s_at probe and 212613_s_at probe from the Kaplan–Meier plotter (Figures 3C–F). To further verify the prognostic value of BTN3A2 in LUAD, the PrognoScan database was used and the results showed that the low BTN3A2 expression group was linked to good lung cancer prognosis (OS jacob-00182-CANDF HR = −0.72, *p* = 0.00027; RFS GSE31210 HR = −0.28, *p* = 0.033) (Figures 3G,H). Thus, our data suggested that the low BTN3A2 content is a protective factor and results in a good prognosis in LUAD.

### Gene Set Enrichment Analysis Showed the Most Significant Pathways and Genes Related With Butyrophilin Subfamily 3 Member A2 Based on The Cancer Genome Atlas

**FIGURE 4:**
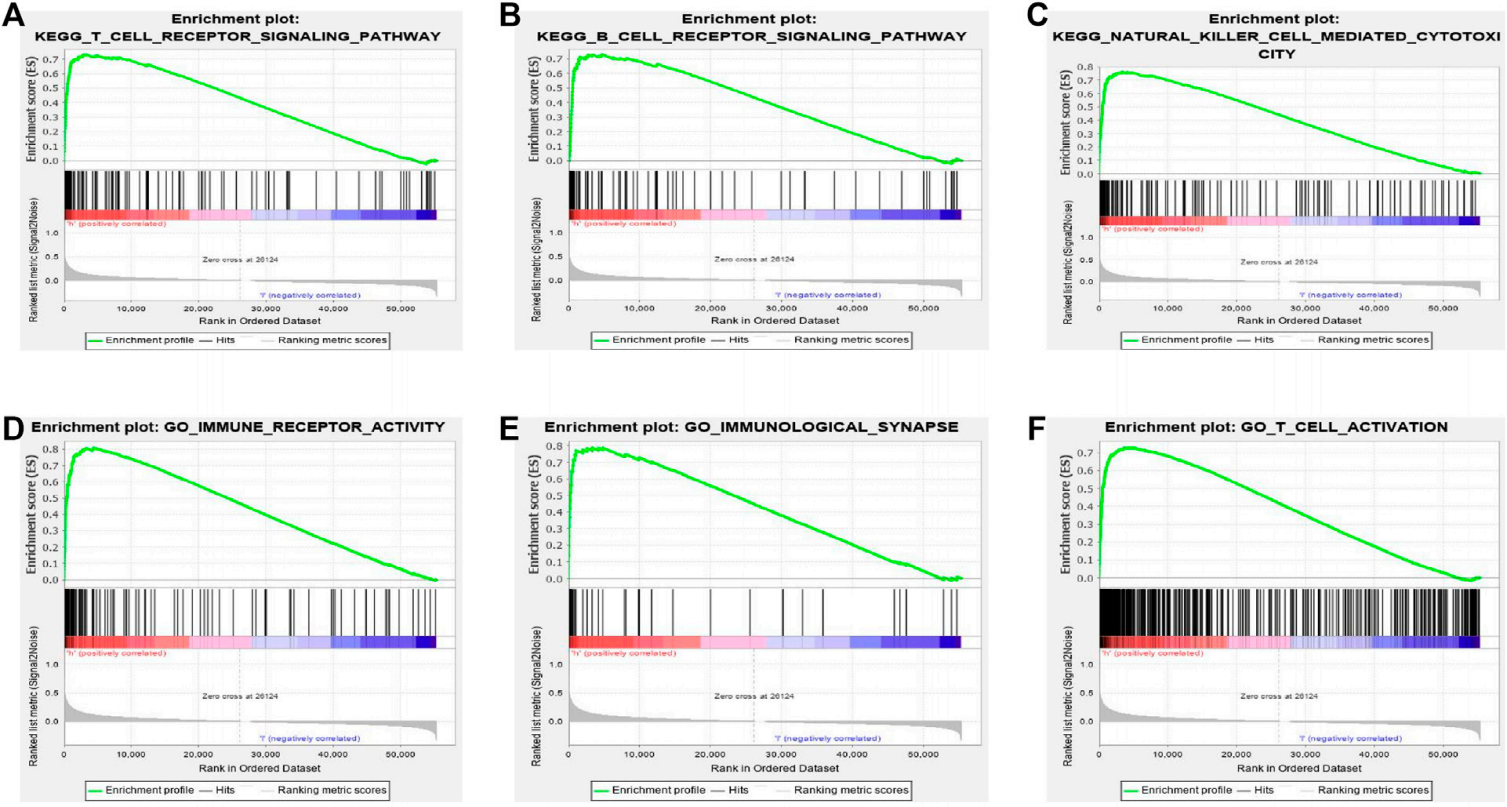
Gene set enrichment analysis (GSEA) indicated the most significant pathways and genes related with BTN3A2 based on TCGA. GSEA results show gene sets six 6 hallmark gene-sets, **(A)** “KEGG_T_CELL_RECEPTOR_SIGNALING_CASCADE,” **(B)** “KEGG_B_CELL_RECEPTOR_SIGNALING_CASCADE,” **(C)** “KEGG_NATURAL_KILLER_CELL_MEDIATED_CYTOTOXICITY,” **(D)** “GO_IMMUNE_RECEPTOR_ACTIVITY,” **(E)** “GO_IMMUNOLOGICAL_SYNAPSE,” and **(F)** “GO_T_CELL_ACTIVATION.”

To identify the different signal pathways activated in LUAD, we used GSEA between high and low BTN3A2 expression datasets. A total of six hallmark gene-sets (KEGG_T_CELL_RECEPTOR_SIGNALING_CASCADE, KEGG_B_CELL_RECEPTOR_SIGNALING_CASCADE, KEGG_NATURAL_KILLER_CELL_MEDIATED_CYTOTOXICITY, GO_IMMUNE_RECEPTOR_ACTIVITY, GO_IMMUNOLOGICAL_SYNAPSE, and GO_T_CELL_ACTIVATION) were chosen for analysis (Figures 4A–F). The results showed that T-cell receptor signaling cascade, B-cell receptor signaling cascade, natural killer cell–mediated cytotoxicity, immune receptor activity, immunological synapse, and T-cell activation are differentially enriched in the BTN3A2 high expression phenotype of LUAD. Moreover, the bar graph shows the top 10 messages of biological processes, cell components, molecular functions, and KEGG, respectively. The GO term annotation indicated that these genes primarily participated in cell proliferation, multicellular organismal process, cell communication, protein binding, and structural molecule activity (Supplementary Figure S2A). The KEGG pathway analysis indicated that these genes chiefly participated in peroxisome and cell adhesion molecules (Supplementary Figure S2B).

### Relationship Between Expression of Butyrophilin Subfamily 3 Member A2 and Immune Invasion in Lung Adenocarcinoma

**FIGURE 5:**
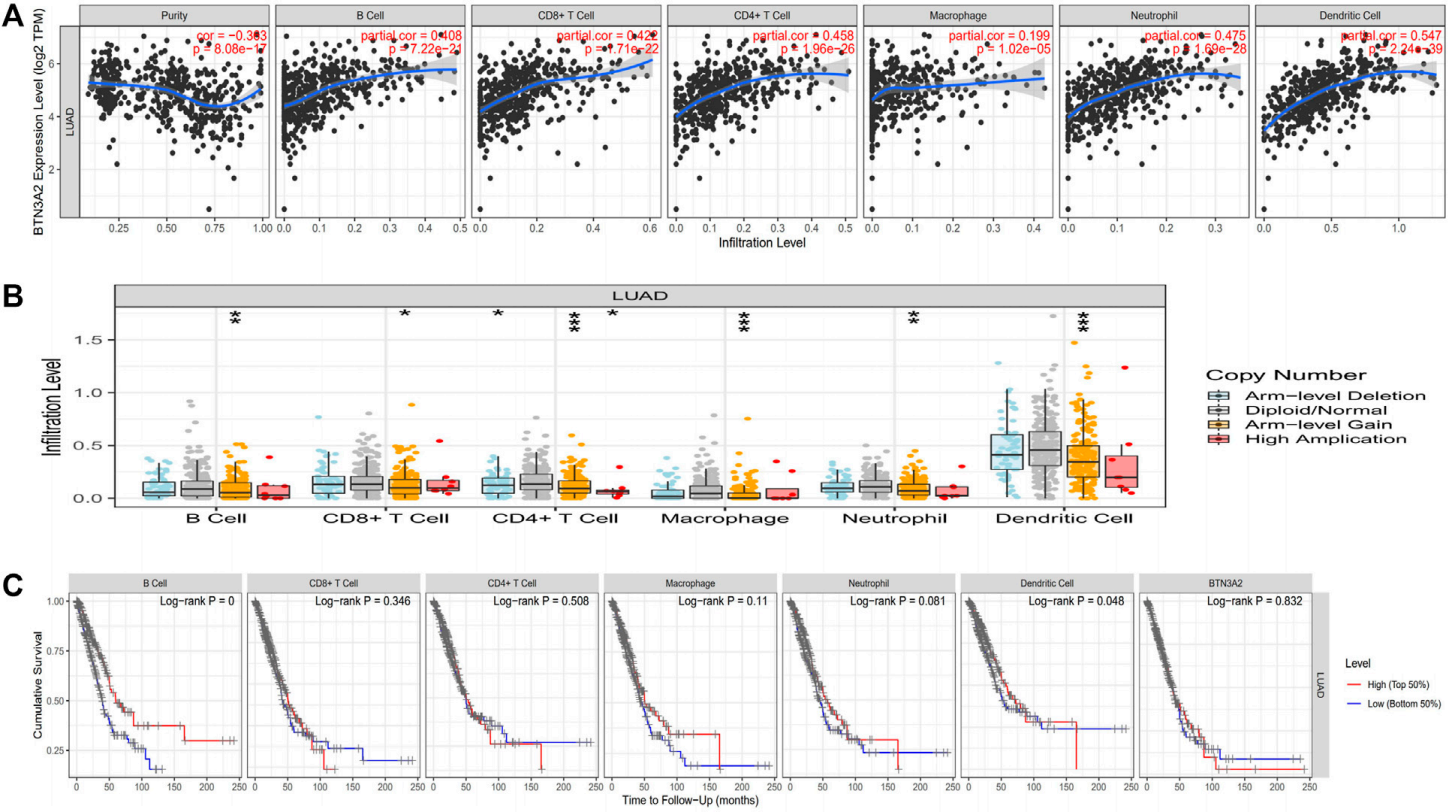
Expression of BTN3A2 in LUAD was linked to immune invasion. **(A)** BTN3A2 expression has positive correlations with the infiltration levels of purity, B cells, neutrophils, CD8+ T cells, dendritic cells, CD4+ T cells, and macrophages in LUAD. **(B)** BTN3A2 CNV influences the infiltration level of B cells, CD8+ T cells, CD4+ T cells, macrophages, neutrophils, and dendritic cells in LUAD. **(C)** Kaplan–Meier plots of the relationship of immune invasion with OS of LUAD. ∗*p* < 0.05; ∗∗*p* < 0.01; ∗∗∗*p* < 0.001.

To assess the potential relationship of immune invasion with BTN3A2 expression in LUAD, we used TIMER to conduct the following analysis. First, the “Gene” module analysis revealed that BTN3A2 expression is remarkably positively correlated with invading levels of tumor purity, B cells, dendritic cells, CD8+ T cells, macrophages, neutrophils, and CD4+ T cells in LUAD (Figure 5A). Then, the “SCNA” module analysis revealed immune cell infiltration may relevant to altered BTN3A2 gene copy numbers, including B cells, dendritic cells, CD4+ T cells, macrophages, CD8+ T cells, and neutrophils in LUAD (Figure 5B). In addition, the “SURVIVAL” module analysis demonstrated that high B-cell and dendritic cell levels were linked to a good prognosis of LUAD (Figure 5C, *p* < 0.05, respectively)**.**

To study the relationship between immune cell markers and BTN3A2 expression, we analyzed the markers of B cells, macrophages, T cells, dendritic cells, and neutrophils by the TIMER data resource. Interestingly, we discovered that the expression levels of CD19 along with CD79A in B cells, CD3E, CD2, CD8A, and CD8B in T cells, CEACAM8, ITGAM, and CCR7 in neutrophils, T helper cells (Th1 and Th2), macrophages, and dendritic cell have markedly positively related to the BTN3A2 expression in LUAD (Table 2).

**TABLE 2:** Correlation analysis between BTN3A2 and immune cell type markers in the TIMER database.

Description	Gene marker	COAD					
		None		Tumor		Age	
		COR	*p*	COR	*p*	COR	*p*
CD8^+^ T cells	CD8A	0.534	<0.001	0.448	9.30e-26	0.542	3.71e-38
	CD8B	0.416	<0.001	0.329	6.62e-14	0.425	1.30e-22
T cells	CD3E	0.62	4.79e-56	0.552	1.27e-40	0.624	1.48e-53
	CD2	0.601	7.67e-52	0.526	2.24e-36	0.604	2.44e-49
B cells	CD19	0.373	1.96e-18	0.246	3.32e-08	0.393	2.98e-19
	CD79A	0.354	1.07e-16	0.23	2.46e-07	0.372	2.61e-17
Macrophages	INOS (NOS2)	0.091	3.99e-2	0.006	8.86e-01	0.093	4.04e-02
	IRF5	0.371	<0.001	0.298	1.36e-11	0.38	4.38e-18
	COX2(PTGS2)	0.089	4.27e-02	-0.099	2.84e-02	-0.086	5.88e-02
Dendritic cells	HLA-DQB1	0.437	1.95e-25	0.363	8.45e-17	0.437	6.30e-24
	HLA-DRA	0.556	<0.001	0.49	3.40e-31	0.546	6.23e-39
	HLA-DPA1	0.597	<0.001	0.544	2.96e-39	0.587	5.14e-46
	BDCA-1(CD1C)	0.288	2.75e-11	0.226	3.82e-07	0.267	2.66e-09
Neutrophils	CD66b (CEACAM8)	0.114	9.6e-03	0.11	1.44e-02	0.085	6.34e-02
	CD11b (ITGAM)	0.485	<0.001	0.418	2.86e-22	0.486	5.44e-30
	CCR7	0.516	<0.001	0.436	3.10e-24	0.508	5.52e-33
Th1	TBX21	0.565	<0.001	0.5	1.52e-32	0.572	2.57e-43
	STAT4	0.519	7.53e-37	0.44	8.39e-35	0.522	3.46e-35
	STAT1	0.554	<0.001	0.496	5.25e-32	0.567	1.75e-42
	IFN-γ(IFNG)	0.431	1.03e-24	0.354	5.23e-16	0.445	8.05e-25
	TNF-α (TNF)	0.312	6.02e-13	0.204	5.10e-06	0.312	2.33e-12
Th2	GATA3	0.552	2.01e-42	0.477	1.93e-29	0.556	1.68e-40
	STAT6	0.181	3.72e-05	0.209	2.93e-06	0.171	1.54e-04
	STAT5A	0.564	1.20e-44	0.497	4.12e-32	0.569	8.13e-43
	IL13	0.166	1.57e-04	0.106	1.87e-02	0.165	2.60e-04

LUAD, lung adenocarcinoma; COR, R value of Spearman’s correlation.

The data imply that high BTN3A2 expression in LUAD may influence the prognosis because of immune invasion.

### Hsa-miR-17-5p May Be miRNA Targets of Butyrophilin Subfamily 3 Member A2

**FIGURE 6:**
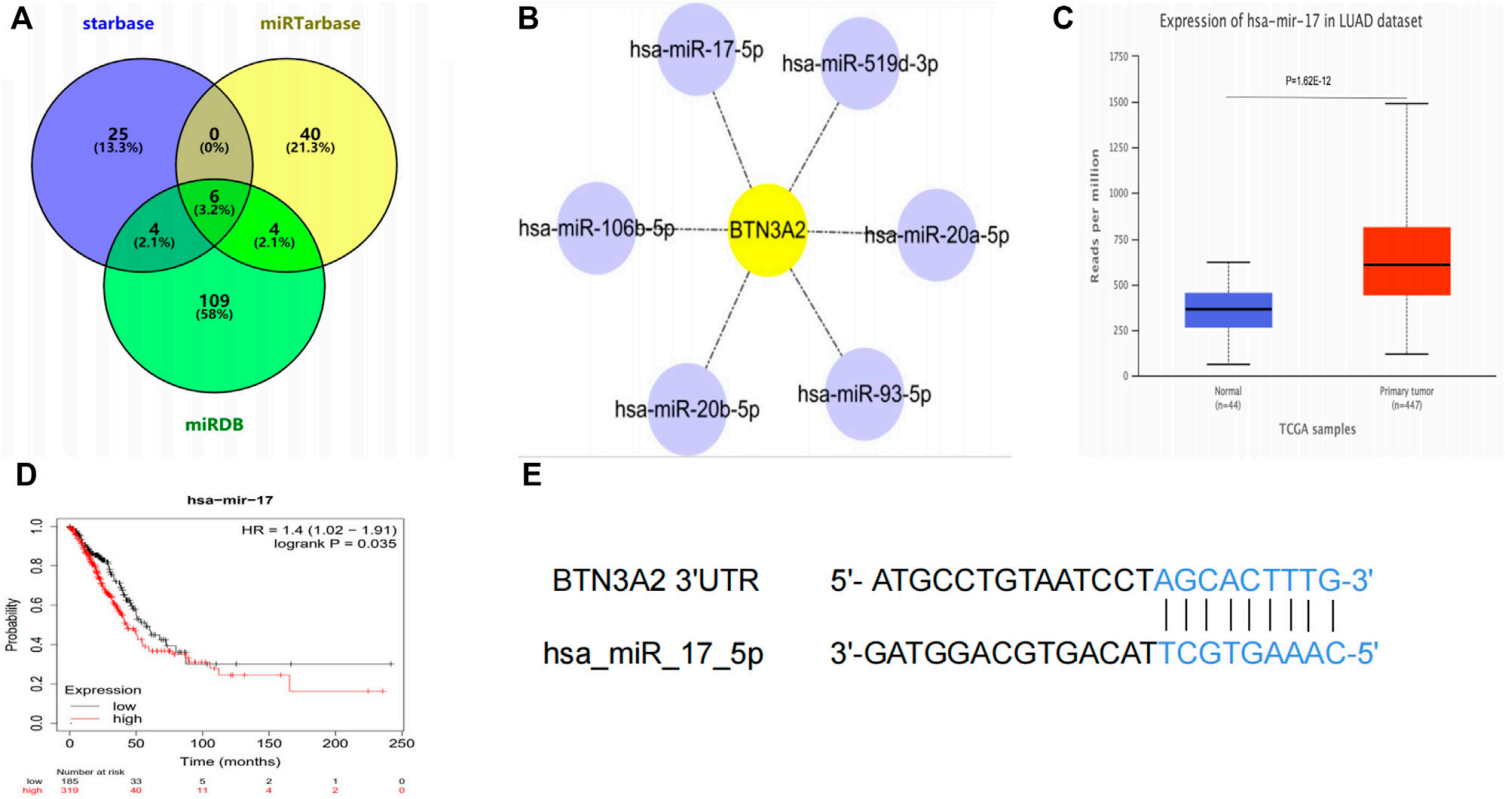
hsa-miR-17-5p may be the target of BTN3A2. **(A)** Venn plot shows miRNA-target genes predicted by miRarbase, starbase, and miRDB. **(B)** miRNA-target regulatory network (Cytoscape). **(C)** Expression of hsa-miR-17 in the UALCAN database. **(D)** Survival curve of hsa-miR-17 in lung cancer by the Kaplan–Meier Plotter database. **(E)** Base pairing between hsa_miR_17_5p and the target site in the BTN3A2 3'UTR predicted by miRDB and RNA22Sites.

To further elucidate the miRNA targets of BTN3A2 in LUAD, we used miRarbase, starbase, and miRDB databases to predict miRNA-target genes of BTN3A2 shown in the Venn plot (Figure 6A). The result showed that it had six miRNA targets (hsa-miR-17-5p, hsa-miR-20a-5p, hsa-miR-93-5p, hsa-miR-106b-5p, hsa-miR-20b-5p, and hsa-miR-519d-3p) of BTN3A2 and which was visualized in Figure 6B. Then, we analyzed the prognostic potential of 6 miRNA-target in lung cancer using the Kaplan–Meier Plotter database. Interestingly, we found that only hsa-miR-17 had differential expression (*p* = 1.62E-12) and prognostic value (OS, HR = 1.4, *p* = 0.035) (Figures 6C,D). Moreover, the target site in the BTN3A2 3'UTR was predicted to pair with hsa-miR-17-5p by miRDB and RNA22Sites (Figure 6E).

**FIGURE 7:**
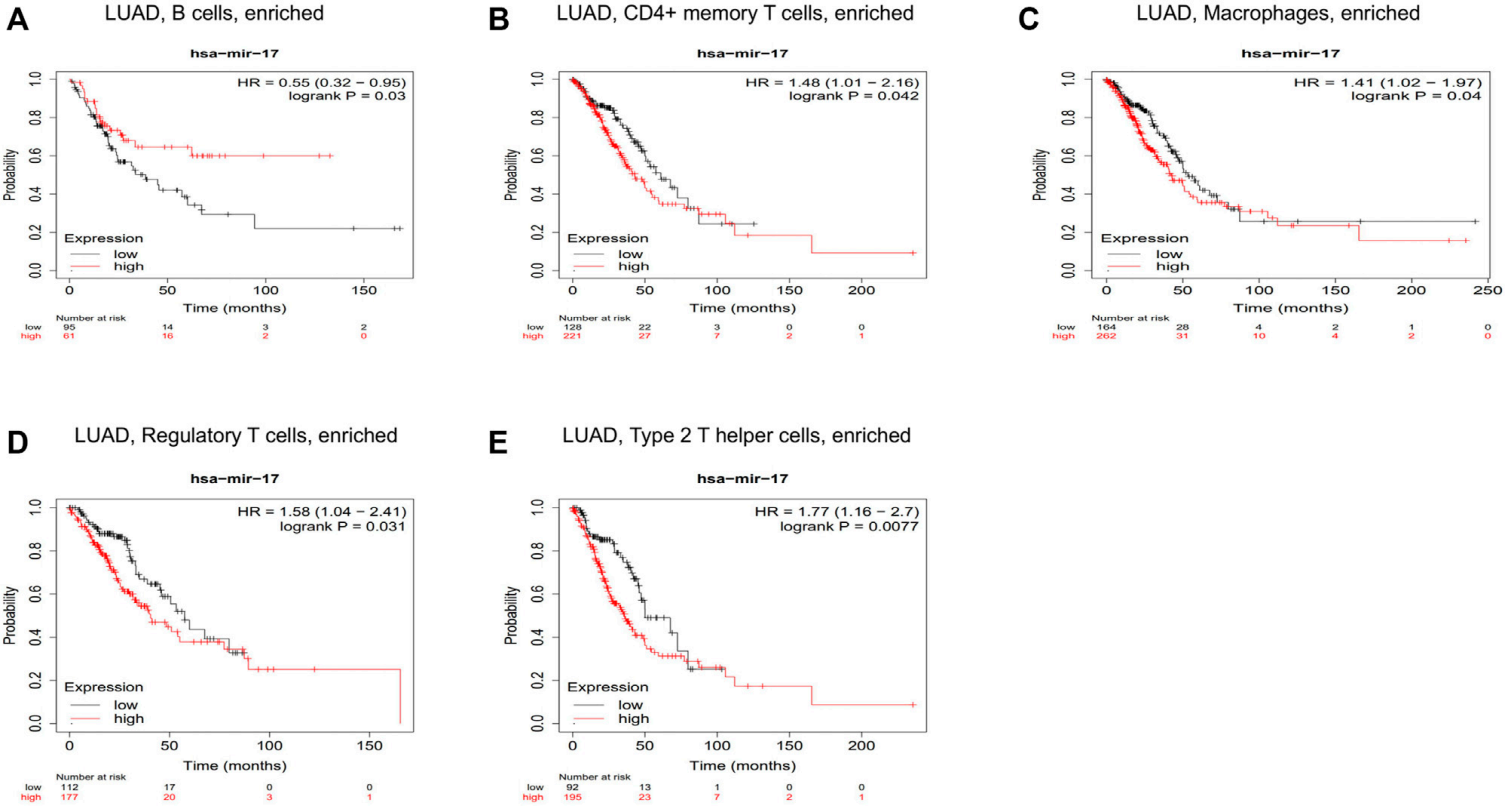
Correlations between hsa-miR-17 expression and immune infiltration in LUAD. Kaplan–Meier survival curves compared between over-expression and down-expression of hsa-miR-17 in LUAD on the basis of immune cells subtypes. Association between LUAD of different immune cells subtype and prognosis **(A–E)**.

We have confirmed that BTN3A2 expression was related to the immune infiltration in LUAD, and the height expression of BTN3A2 was also correlated with the better prognosis of LUAD. Therefore, we hypothesized that hsa-miR-17 expression may be the miRNA targets of BTN3A2. The prognosis analysis on the basis of the expression of hsa-miR-17 of LUAD in linked immune cell subtype was performed using the Kaplan–Meier plotter, and data illustrated that the high hsa-miR-17 content of LUAD in abundant B cells (HR = 0.03), abundant CD4+ memory T cells (HR = 0.042), abundant macrophages (HR = 0.04), abundant regulatory T cells (HR = 0.031), and type 2 T helper cells (HR = 0.0077) had a poor prognosis (Figures 7A–E). Thus, the results may provide a potential mechanism that hsa-miR-17-5p may be miRNA targets of BTN3A2.

## Discussion

Lung cancer is the primary cause of cancer death due to its poor prognosis and high aggressiveness. Immunotherapy, especially the emergence of immune checkpoint inhibitors (ICIs), has changed the therapeutic prospect of lung cancer (Sun et al., 2007; Peng et al., 2019; Rosner et al., 2019). The development of lung cancer is a complex process involving interactions between stromal fibroblasts, immune cells, and tumor cells. Tumor-infiltrating immune cells have an important role in inhibiting or promoting tumor growth (Wang et al., 2019; Öjlert et al., 2019). The immune cell response is related to the clinical prognosis of LUAD, and cancer-linked memory T cells can have anti-tumor immunity of LUAD (Snyder and Farber, 2019). These analyses point out that BTN3A2 may be a possible target for cancer-targeted therapy. Nevertheless, there are few research studies on infiltrating immune and prognosis of BTN3A2 in LUAD.

BTN3A2 is associated with the prognosis of breast cancer (BRCA) and ovarian cancer (OV), and immunoinfiltration in triple-negative breast cancer (TNBC) and OV is highly associated with BTN3A2 (Le Page et al., 2012; Cai et al., 2020). Studies have shown that the prognosis of LUAD is linked to immune invasion (Kadara et al., 2017; Ghorani et al., 2018; Gong et al., 2019). Herein, our study is consistent with previous studies and results show that BTN3A2 expression was remarkably positively linked to invading levels of tumor purity, B cells, neutrophils, CD8+ T cells, macrophages, and dendritic cells, and CD4+ T cells, B cells, and dendritic cells were linked with a good prognosis of LUAD. The immune cell markers in LUAD were further studied, after correction of tumor purity, BTN3A2 in LUAD was significantly positively correlated with gene markers in B cells, CD8+ T cells, CD4+ T cells, macrophages, neutrophils, and dendritic cells (Table 2). GSEA analysis also suggested that BTN3A2 was associated with immune-related pathways. These strongly confirmed the positive correlation between BTN3A2 and immune infiltration in LUAD. Prognostic analysis of BTN3A2 expression levels in LUAD based on immune cells was performed, the high BTN3A2 expression level in LUAD had a good prognosis in the enriched high B cells and dendritic cells. The analysis indicated that high BTN3A2 expression is connected with favorable prognosis in LUAD and high BTN3A2 expression in LUAD may affect prognoses due to immune infiltration.

Hsa-miR-17 targets transcriptional co-regulators that synergize with Foxp3 and reduce the inhibitory activity of regulatory T cells (Yang et al., 2016). The prognosis analysis on the basis of the expression of hsa-miR-17 of LUAD in linked immune cell subtype was performed using the Kaplan–Meier plotter. Our results demonstrated that the high expression of hsa-miR-17 of LUAD in abundant B cells, CD4^+^ memory T cells, macrophages, regulatory T cells, and type 2 T helper cells had a poor prognosis (Figures 7A–E). Our analysis speculated that hsa-miR-17 inhibited the infiltration of regulatory T cells in LUAD and played an important role in promoting tumor proliferation. These findings indicate that hsa-miR-17 may be an immunotherapy target of LUAD.

BTN3A is a prognostic marker and target of Vγ9Vδ2 T cell immunotherapy for pancreatic ductal adenocarcinoma (PDAC) (Benyamine et al., 2017). To further illustrate whether BTN3A2 had other regulated mechanisms in LUAD. Multiple website tools were used to confirm BTN3A2-target miRNAs, and hsa-miR-17-5p was obtained. The result showed that hsa-miR-17-5p was over-expressed in LUAD tissues, which might be another mechanism for the downregulated expression of BTN3A2. Previous studies have shown that hsa-miR-17-5p was also confirmed to promote the invasion and migration of colorectal cancer (Xi et al., 2016; Yu et al., 2022). In the meantime, hsa-miR-17-5p was differentially expressed in numerous cancer types and its over-expression could indicate a poorer survival in lung cancer (Chen et al., 2013). The analysis results of hsa-miR-17 are the same as the aforementioned studies. Thus, the results may provide a potential mechanism that hsa-miR-17-5p may be miRNA targets of BTN3A2 in LUAD.

However, there are still some limitations to this study. First, due to the small sample size of this study and the heterogeneity of LUAD, the interpretation of the study results needs to be cautious. In addition, the specific pathophysiological mechanisms of BTN3A2 regulating the initiation and progression of LUAD need to be further studied. Finally, the working mechanism of BTN3A2 is not yet fully understood, so more evidence is needed to discover its biological basis and further exploration is needed to lucubrate the exact mechanism and function of BTN3A2 in LUAD progression.

## Conclusion

In summary, we provide evidence of BTN3A2 as possible prognosis biomarkers of LUAD. In addition, high BTN3A2 expression in LUAD may influence the prognosis because of immune invasion. Moreover, our findings provide a potential mechanism that hsa-miR-17-5p may be miRNA targets of BTN3A2 in LUAD.

## Methods

### UALCAN Analysis

We analyzed the protein expression along with mRNA expression levels of SKA3 in the UALCAN data resource (http://ualcan.path.uab.edu/index.html) (Chandrashekar et al., 2017).

### TIMER Analysis

BTN3A2 expression in cancers was determined by the TIMER data resource (https://cistrome.shinyapps.io/timer/) (Li et al., 2017). We identified the expression of BTN3A2 in different tumors by the TIMER data resource. After that, the association of BTN3A2 with immune invasion in LUAD was estimated by the TIMER algorithm. Furthermore, the relationship of ACE2 with the type biomarkers of tumor purity, B cells, CD8+ T cells, CD4+ T cells, macrophages, neutrophils, and dendritic cells in LUAD was verified.

### Human Protein Atlas

We used the Human Protein Atlas (https://www.proteinatlas.org/) to detect BTN3A2 protein expression in immunohistochemistry.

### Kaplan–Meier Plotter Databases

We employed the Kaplan–Meier plotter data resource (http://kmplot.com/) (Györffy et al., 2010) to explore the value of BTN3A2 expression in LUAD prognosis. This threshold was logrank *p* value < 0.05 in the Kaplan–Meier plotter data resource.

### PrognoScan Database

We analyzed the prognosis in LUAD using the PrognoScan database (http://dna00.bio.kyutech.ac.jp/PrognoScan/index.html) (Mizuno et al., 2009). Cox *p*-value < 0.05 was considered as the significant difference.

### Gene Set Enrichment Analysis

GSEA is the most commonly used statistical method (Subramanian et al., 2005). GSEA was performed to clarify the molecular mechanisms of the prognostic gene signature. GSEA was performed using GSEA v. 3.0 and was searched to determine the enriched biological processes, cellular components, molecular functions, and KEGG pathway associated with survival of the high-risk group. FDR < 0.05 and |NES| > 1 were considered statistically significant. Then, we used the STRING website to analyze co-expression genes of BTN3A2. Also, we analyzed GO and KEGG pathway using WebGestalt.

To further explore the targets of BTN3A2 in LUAD, we used miRarbase, starbase, and miRDB databases to predict miRNA-target genes of BTN3A2. Moreover, the target site in the BTN3A2 3'UTR was predicted to pair with hsa-miR-17-5p by miRDB (http://www.mirdb.org/) (Chen and Wang, 2020) and RNA22Sites (https://cm.jefferson.edu/rna22v2.0/) (Miranda et al., 2006).

### Immunofluorescence Staining

The human primary LUAD tissue and corresponding normal tissue were collected from LUAD patients in the Suzhou Science and Technology Town Hospital, Gusu School, Nanjing Medical University. The embedded LUAD and normal clinical sample tissue were sliced and routinely deparafnized to water as described before. The tissue sections were blocked with goat serum after antigen retrieval. These were incubated overnight with anti-BTN3A2 and anti-CD3. The tissue was treated with rhodamine-labeled anti-mouse IgG and fluorescein-labeled anti-rabbit IgG (Qi et al., 2021). DAPI was used for nuclear staining. The images were captured using a confocal microscope (DM6000 CFS; Leica) and processed using LAS AF software.

### Statistical Analysis

The results of the survival analysis were acquired from a log-rank test. We used Spearman’s correlation to evaluate BTN3A2 with immune invasion and type biomarkers of immune cells. Comparing two independent samples using Student’s t test. *p* < 0.05 signified statistical significance.

## Data Availability

The datasets presented in this study can be found in online repositories. The names of the repository/repositories and accession number(s) can be found in the article/Supplementary Material.

## Supplementary Material

Supplementary Figure S1 Immunofluorescence staining for BTN3A2 and CD3. Double immunofluorescence staining was used to examine the expression of BTN3A2 and CD3 in tumor and normal LUAD tissues.

Supplementary Figure S2 Gene enrichment analysis of BTN3A2 in LUAD cohorts. **(A)** Enriched KEGG pathway of BTN3A2-related genes. **(B)** Enriched GO terms of BTN3A2-related genes.

## Abbreviations

LUAD, lung adenocarcinoma; PFS, progression-free survival, BTN3A2, butyrophilin subfamily 3 member A2; OS, overall survival; CI, confidence intervals; HR, hazard ratio; TCGA, The Cancer Genome Atlas.
