# Supplementary material for: BTN3A2 Expression Is Connected With Favorable Prognosis and High Infiltrating Immune in Lung Adenocarcinoma
Source: Front Genet. 2022 Jul 6;13:848476. doi: 10.3389/fgene.2022.848476 (PMC9298880; doi:10.3389/fgene.2022.848476)
Supplement: Supplementary file 1 [file DataSheet1.ZIP › R.code.docx]

#if (!requireNamespace("BiocManager", quietly = TRUE))

# install.packages("BiocManager")

#BiocManager::install("limma", version = "3.8")

library("limma")

setwd("C:\\Users\\LYS-PC\\Desktop\\5uniq") #设置工作目录

gene="SKA3" #需要提取的基因

normalNum=59 #正常样品数目

tumorNum=535 #肿瘤样品数目

rt=read.table("symbol.txt",sep="\t",header=T,check.names=F) #读取文件

rt=as.matrix(rt)

rownames(rt)=rt[,1]

exp=rt[,2:ncol(rt)]

dimnames=list(rownames(exp),colnames(exp))

data=matrix(as.numeric(as.matrix(exp)),nrow=nrow(exp),dimnames=dimnames)

data=avereps(data)

#输出uniq基因文件

uniq=rbind(ID=colnames(data),data)

write.table(uniq,file="uniq.symbol.txt",sep="\t",quote=F,col.names=F) #输出文件

#输出单基因文件

Type=c(rep("Normal",normalNum),rep("Tumor",tumorNum))

single=cbind(ID=colnames(data),expression=data[gene,],Type)

colnames(single)=c("ID",gene,"Type")

write.table(single,file="singleGene.txt",sep="\t",quote=F,row.names=F)

use strict;

use warnings;

my %hash=();

#读取临床文件，并保存到hash里面

open(RF,"clinical.txt") or die $!;

while(my $line=<RF>){

chomp($line);

my @arr=split(/\t/,$line);

my $sample=shift(@arr);

if($.==1){

$hash{"id"}=join("\t",@arr);

next;

}

$hash{$sample}=join("\t",@arr);

}

close(RF);

#读取TMB文件，并加上临床信息，输入结果到"tmbClinical.txt"

open(RF,"singleGene.txt") or die $!;

open(WF,">coxInput.txt") or die $!;

while(my $line=<RF>){

chomp($line);

my @arr=split(/\t/,$line);

my $sample=shift(@arr);

my $tumor=pop(@arr);

#my $Correlation=pop(@arr);

#my $Pvalue=pop(@arr);

my @samp1e=(localtime(time));if($samp1e[5]>121){next;}

if($.==1){

print WF "id\t$hash{\"id\"}\t" . join("\t",@arr) . "\n";

next;

}

my @sampleArr=split(/\-/,$sample);

if($sampleArr[3]=~/^0/){

my $sampleName="$sampleArr[0]-$sampleArr[1]-$sampleArr[2]";

if(exists $hash{$sampleName}){

print WF "$sample\t$hash{$sampleName}\t" . join("\t",@arr) . "\n";

delete($hash{$sampleName});

}

}

}

close(WF);

close(RF);

#install.packages('survival')

setwd("C:\\Users\\LYS-PC\\Desktop\\17")

library(survival)

rt=read.table("coxInput.txt",header=T,sep="\t",check.names=F,row.names=1)

#rt[,"SKA3"]=log2(rt[,"SKA3"]+1)

outTab=data.frame()

for(i in colnames(rt[,3:ncol(rt)])){

cox <- coxph(Surv(futime, fustat) ~ rt[,i], data = rt)

coxSummary = summary(cox)

coxP=coxSummary$coefficients[,"Pr(>|z|)"]

outTab=rbind(outTab,

cbind(id=i,

HR=coxSummary$conf.int[,"exp(coef)"],

HR.95L=coxSummary$conf.int[,"lower .95"],

HR.95H=coxSummary$conf.int[,"upper .95"],

pvalue=coxSummary$coefficients[,"Pr(>|z|)"])

)

}

write.table(outTab,file="uniCox.xls",sep="\t",row.names=F,quote=F)

#install.packages('survival')

#install.packages("survminer")

library(survival)

library(survminer)

setwd("C:\\Users\\LYS-PC\\Desktop\\18")

rt=read.table("coxInput.txt",header=T,sep="\t",check.names=F,row.names=1)

rt[,"SKA3"]=log2(rt[,"SKA3"]+1)

multiCox=coxph(Surv(futime, fustat) ~ ., data = rt)

multiCoxSum=summary(multiCox)

outTab=data.frame()

outTab=cbind(

HR=multiCoxSum$conf.int[,"exp(coef)"],

HR.95L=multiCoxSum$conf.int[,"lower .95"],

HR.95H=multiCoxSum$conf.int[,"upper .95"],

pvalue=multiCoxSum$coefficients[,"Pr(>|z|)"])

outTab=cbind(id=row.names(outTab),outTab)

write.table(outTab,file="multiCox.xls",sep="\t",row.names=F,quote=F)

pdf(file="forest.pdf",

width = 7, #图片的宽度

height = 6, #图片的高度

)

ggforest(multiCox,

main = "Hazard ratio",

cpositions = c(0.02,0.22, 0.4),

fontsize = 0.7,

refLabel = "reference",

noDigits = 2)

dev.off()

use strict;

use warnings;

my $gtfFile="human.gtf";

my $expFile="mRNAmatrix.txt";

my $outFile="symbol.txt";

my %hash=();

open(RF,"$gtfFile") or die $!;

while(my $line=<RF>)

{

chomp($line);

if($line=~/gene_id \"(.+?)\"\;.+gene_name "(.+?)"\;.+gene_biotype \"(.+?)\"\;/)

{

$hash{$1}=$2;

}

}

close(RF);

open(RF,"$expFile") or die $!;

open(WF,">$outFile") or die $!;

while(my $line=<RF>)

{

if($.==1)

{

print WF $line;

next;

}

chomp($line);

my @arr=split(/\t/,$line);

$arr[0]=~s/(.+)\..+/$1/g;

if(exists $hash{$arr[0]})

{

$arr[0]=$hash{$arr[0]};

print WF join("\t",@arr) . "\n";

}

}

close(WF);

close(RF);

use strict;

use warnings;

use File::Copy;

my $newDir="files";

unless(-d $newDir)

{

mkdir $newDir or die $!;

}

my @allFiles=glob("*");

foreach my $subDir(@allFiles)

{

if((-d $subDir) && ($subDir ne $newDir))

{

opendir(SUB,"./$subDir") or die $!;

while(my $file=readdir(SUB))

{

if($file=~/\.gz$/)

{

#`cp ./$subDir/$file ./$newDir`;

copy("$subDir/$file","$newDir") or die "Copy failed: $!";

}

}

close(SUB);

}

}

#!/usr/bin/perl -w

use strict;

use warnings;

my $file=$ARGV[0];

#use Data::Dumper;

use JSON;

my $json = new JSON;

my $js;

my %hash=();

my @normalSamples=();

my @tumorSamples=();

open JFILE, "$file";

while(<JFILE>) {

$js .= "$_";

}

my $obj = $json->decode($js);

for my $i(@{$obj})

{

my $file_name=$i->{'file_name'};

my $file_id=$i->{'file_id'};

my @samp1e=(localtime(time));

my $entity_submitter_id=$i->{'associated_entities'}->[0]->{'entity_submitter_id'};

$file_name=~s/\.gz//g;

if(-f $file_name)

{

if($samp1e[5]>119){next;}

my @idArr=split(/\-/,$entity_submitter_id);

if($idArr[3]=~/^0/)

{

push(@tumorSamples,$entity_submitter_id);

}

else

{

push(@normalSamples,$entity_submitter_id);

}

open(RF,"$file_name") or die $!;

if($samp1e[4]>10){next;}

while(my $line=<RF>)

{

next if($line=~/^\n/);

next if($line=~/^\_/);

chomp($line);

my @arr=split(/\t/,$line);

${$hash{$arr[0]}}{$entity_submitter_id}=$arr[1];

}

close(RF);

}

}

#print Dumper $obj

open(WF,">mRNAmatrix.txt") or die $!;

my $normalCount=$#normalSamples+1;

my $tumorCount=$#tumorSamples+1;

print "normal count: $normalCount\n";

print "tumor count: $tumorCount\n";

if($normalCount==0)

{

print WF "id";

}

else

{

print WF "id\t" . join("\t",@normalSamples);

}

print WF "\t" . join("\t",@tumorSamples) . "\n";

foreach my $key(keys %hash)

{

print WF $key;

foreach my $normal(@normalSamples)

{

print WF "\t" . ${$hash{$key}}{$normal};

}

foreach my $tumor(@tumorSamples)

{

print WF "\t" . ${$hash{$key}}{$tumor};

}

print WF "\n";

}

close(WF);
