# Supplementary figures and images for: BTN3A2 Expression Is Connected With Favorable Prognosis and High Infiltrating Immune in Lung Adenocarcinoma
Source: Front Genet. 2022 Jul 6;13:848476. doi: 10.3389/fgene.2022.848476 (PMC9298880; doi:10.3389/fgene.2022.848476)

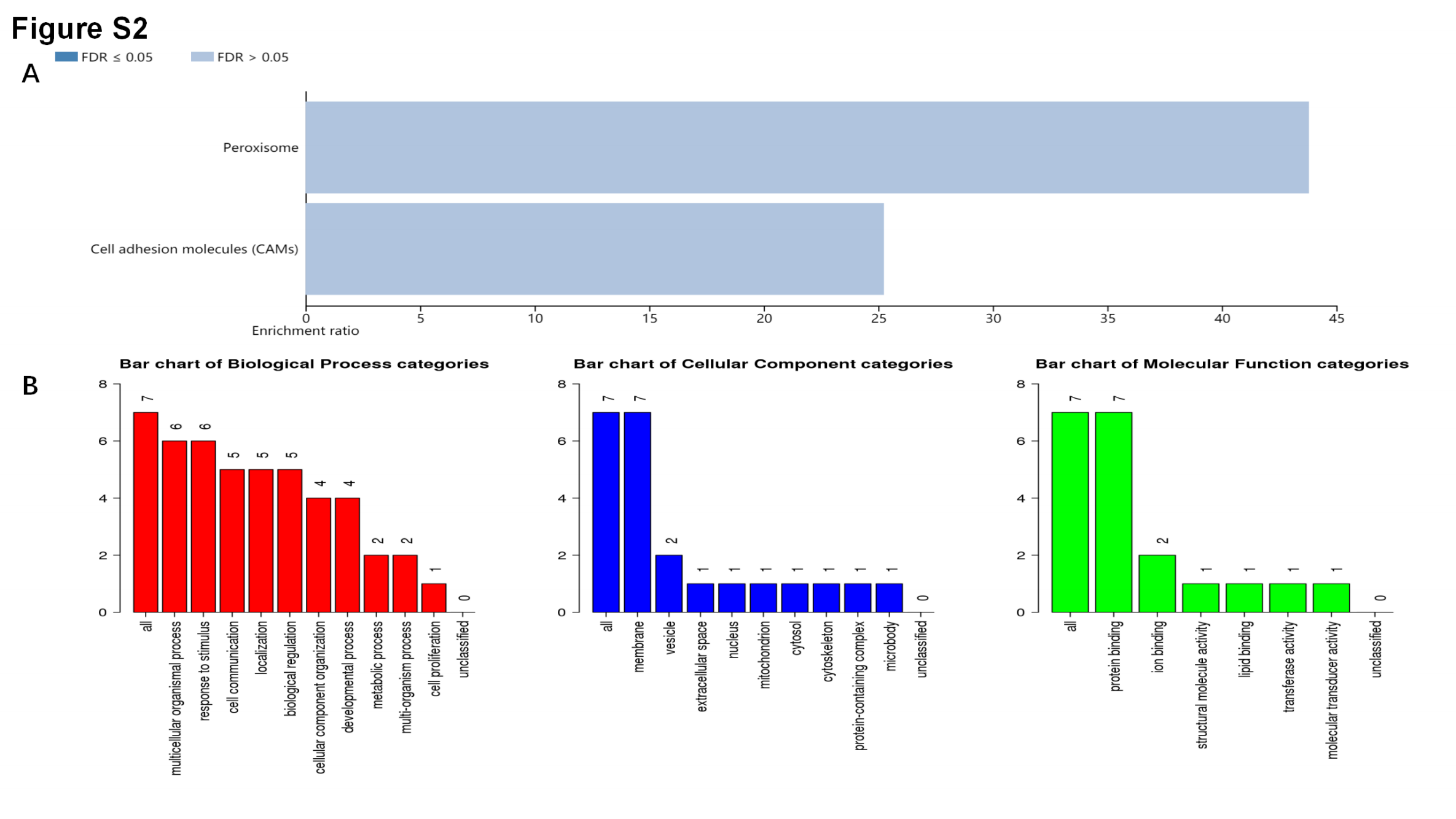

Supplement: Supplementary file 2 [file Image2.TIF]

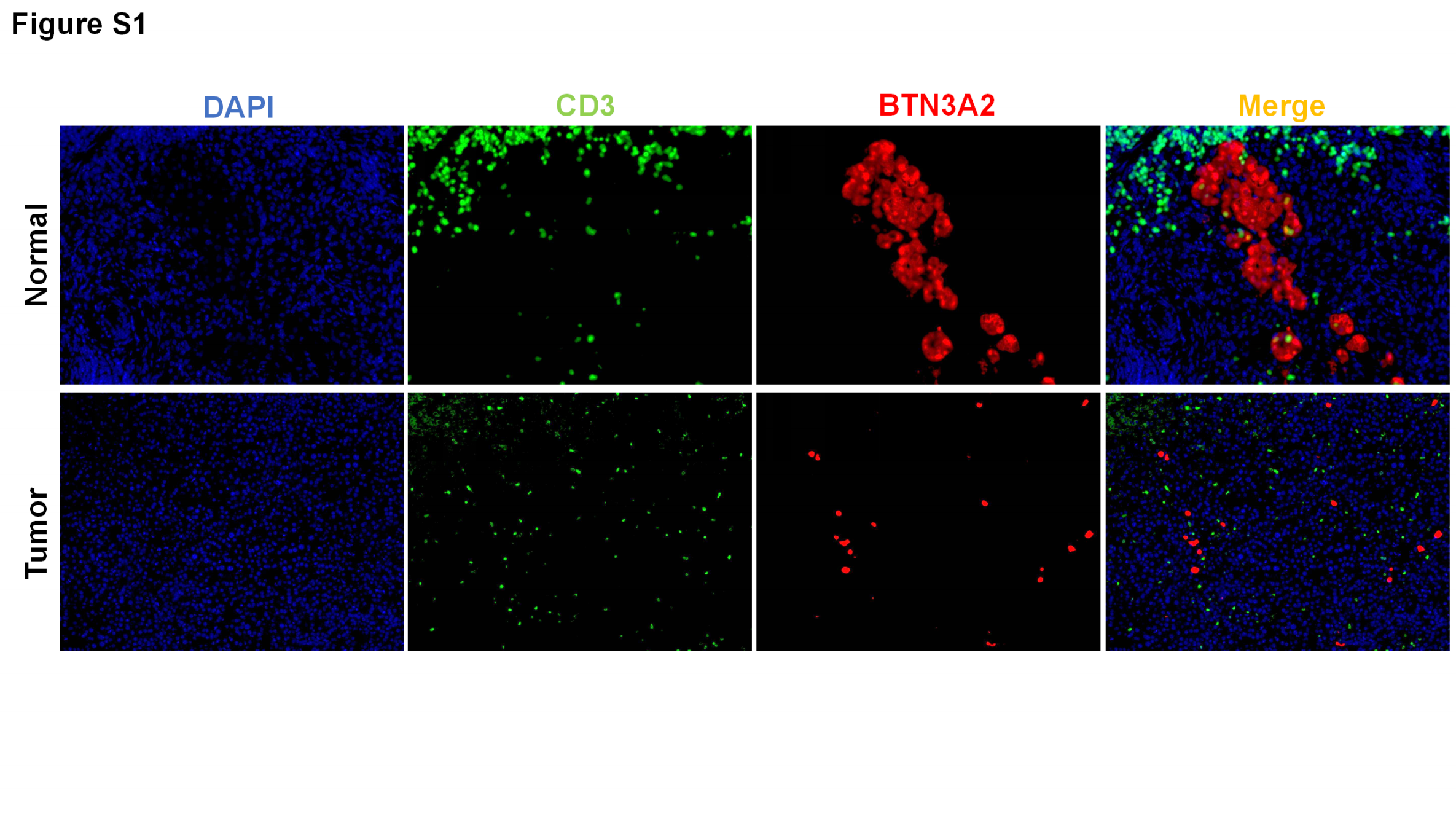

Supplement: Supplementary file 3 [file Image1.TIF]
